# Elevated Numbers of Circulating Very Small Embryonic-Like Stem Cells (VSELs) and Intermediate CD14++CD16+ Monocytes in IgA Nephropathy

**DOI:** 10.1007/s12015-018-9840-y

**Published:** 2018-07-19

**Authors:** Andrzej Eljaszewicz, Katarzyna Kleina, Kamil Grubczak, Urszula Radzikowska, Paula Zembko, Paulina Kaczmarczyk, Marlena Tynecka, Karolina Dworzanczyk, Beata Naumnik, Marcin Moniuszko

**Affiliations:** 1 Department of Regenerative Medicine and Immune Regulation, Medical University of Bialystok, ul. Waszyngtona 13, 15-269 Bialystok, Poland; 2 1st Department of Nephrology with Dialysis Unit, Medical University of Bialystok, Bialystok, Poland; 3 Department of Allergology and Internal Medicine, Medical University of Bialystok, Bialystok, Poland

**Keywords:** VSELs, Monocytes, EPCs, HSCs, IgA nephropathy, SDF-1, sCD163, Ang-1, Ang-2, Regeneration

## Abstract

IgA nephropathy (IgAN) is recognized as most frequent form of primary glomerulonephritis worldwide. IgAN is associated with renal degradation occurring due to irreversible pathological changes leading to glomerulosclerosis and interstitial fibrosis. It remains poorly understood whether and to what extent these changes are followed by the activation of regenerative mechanisms. Therefore, in this study we aimed to evaluate regenerative potential of IgAN patients by quantitating the frequencies of several stem cell types, namely circulating very small embryonic-like stem cells (VSELs), hematopoietic stem cells (HSCs), endothelial progenitor cells (EPCs) as well as different monocyte subsets with varying maturation and angiopoietic potential. Moreover, we analyzed whether changes in stem cell and monocyte frequencies were related to alterations of several chemotactic factors (stromal derived-factor (SDF-1), angiopoietin-1 (Ang-1) and angiopoietin-2 (Ang-2)) and a marker of monocyte/macrophage activation, namely soluble form of CD163 receptor (sCD163). We showed that IgAN patients presented with enhanced levels of VSELs, but not other stem cell types. We also demonstrated significantly elevated numbers of intermediate monocytes known for their M2-like properties as well as high angiopoietic potential and CD163 expression. This finding was accompanied by detection of elevated sCD163 plasma levels in IgAN patients. Taking together, we demonstrated here that IgAN is associated with selective mobilization of VSELs and increased maturation of monocytes towards M2-like and angiopoietic phenotype. These findings contribute to better understanding of the role of regenerative mechanisms in the pathogenesis of chronic inflammation in the course of IgAN.

## Introduction

IgA nephropathy (IgAN) is recognized as most frequent form of primary glomerulonephritis worldwide. Diagnosis is established exclusively by kidney biopsy where the pathognomonic finding on immunofluorescence microscopy is the presence of mesangial deposits of IgA antibodies. Exact mechanisms leading to development of IgAN are unclear and despite available treatment options, slow progression to end stage renal disease occurs in up to 50% of affected patients [1]. Therefore, there is still a substantial need for better understanding of mechanisms involved in IgAN pathogenesis and renal regeneration processes.

However, to date, it remains elusive whether pathological changes observed in IgAN are somehow counteracted by the activation of any regenerative mechanisms related to stem cells or other cells with regenerative potential. Stem cells with highest regenerative potential such as hematopoietic stem cells (HSCs), very small embryonic like cells (VSELs), epithelial progenitor cells (EPCs) and mesenchymal stem cells (MSCs) can be found in bone marrow [2, 3]. Importantly, several reports indicated that bone marrow derived stem cells may support regeneration of such distant tissues as kidney [4]. These stem cell subsets can differentiate into tubular epithelial cells, mesangial cells, endothelial cells and podocytes [5–9]. Notably, in several mouse models of renal injury, including folic acid induced acute tubular injury and ischemia-reperfusion injury, bone marrow derived cells showed reparative capacity [10–12]. On the other hand, bone marrow derived cells may function as local regulators of inflammatory processes directly via cell-to-cell interaction and indirectly by release of anti-inflammatory mediators, including cytokines, chemokines and growth factors [13, 14]. In a consequence, they can act as orchestrators of innate and adaptive immune response within injured tissue. This can lead to increased migration and modulation of the function of leukocytes, including monocytes/macrophages [15–17]. In fact, several populations of mononuclear cells such as M2 macrophages and intermediate monocytes characterized by CD14++CD16+ phenotype can support stem cells in some regenerative actions, mostly those related to reconstruction of injured vessels [18, 19]. These mononuclear cells were shown to support regeneration process mostly by regulation of angiogenesis and the release of several growth factors [18, 20, 21]. To date, however, our understanding of the mechanisms of renal regeneration in humans remains limited. Therefore, in this study, we wished to evaluate regenerative potential of IgAN patients by enumerating different stem cell subsets as well as monocyte subsets with varying reparatory capacity.

## Materials and Methods

### Patients

Protocol of the study adhered to the principles of the Declaration of Helsinki and was approved by the local ethics committee. All patients provided informed consent.

Patients were recruited in outpatient clinic of Department of Nephrology. Inclusion criteria were following: clinically stable, biopsy-proven IgAN in stages 1–4 of chronic kidney disease. Exclusion criteria were following: eGFR<15 ml/min/1,73 m2 (CKD stage 5) as the authors assumed irreversibility of kidney damage, chronic or acute infection or other inflammatory disease, neoplasmatic disease, immunosuppressive treatment at the moment of recruitment and in the 12 months preceding recruitment.

All patients were Caucasian. Mean age was 44 years. There were 12 females in study group. Majority of the patients received RAAS blockade in maximal dose (ACE-I/ARB). 19 (73%) persons had well-controlled hypertension. 16 (62%) patients received immunosuppressive (steroids) treatment in the past. Full clinical characteristic of the patients is given in Table 1.

**Table 1:** Clinical characteristic of study population

PARAMETER	IGAN	CONTROLS
N	26	17
Age mean (SD)	44 (12)	43.4 (10.3)
Gender (Female/Male)	12/14	11/6
Stages of CKD (n/%)		
Stage 1	7 (27)	
Stage 2	6 (23)	
Stage 3	6 (23)	
Stage 4	7 (27)	
Patients receiving RAAS blockade in maximal dose (n/%)	18 (69)	
Patients without any RAAS blockade (n/%)	2 (8)	
History of immunosuppressive treatment (n/%)	16 (62)	
Hypertension (n/%)	19 (73)	
Laboratory parameters (mean, SD)		
Creatinine (mg/dl)	1.72 (1.04)	
eGFR (ml/min/1,73m^2^)	60.81 (37.48)	
CRP (mg/l)	3.08 (5.27)	
WBC (10^9^/l)	7.5 (1.84)	
RBC (10^9^/l)	4.48 (0.60)	
HGB (g/dl)	13.61 (1.77)	
PLT (×10 [9]/l	213.5 (48.89)	
Urine protein to creatinine ratio (uPCR) (mean,SD)	1.22 (1.43)	
Presence of hematuria (n/% of pts)	18 (69)	

The healthy controls were 17 Caucasian volunteers, 6 men and 11 women, and the mean age was 43.4 ± 10.3 years (range, 29–63 years).

Fasting peripheral blood samples were obtained from all recruited patients and controls and immediately transferred to the laboratory.

### Flow Cytometry

**Fig. 1:**
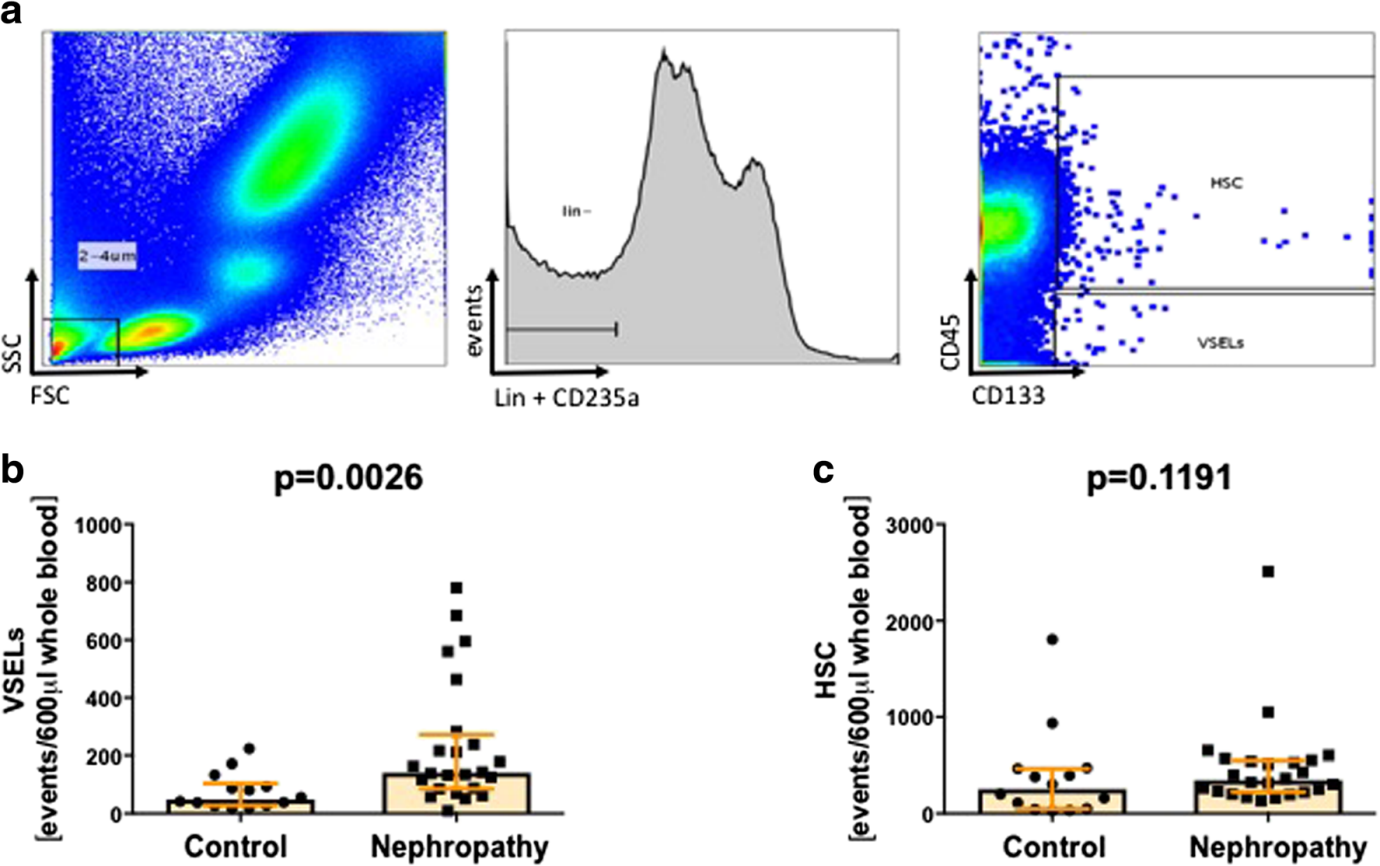
VSELs, but not HSCs are effectively mobilized to the periphery in IgA nephropathy patients. Representative gating strategy for flow cytometric enumeration of VSELs and HSCs (**a**). Summary of flow cytometry analyses of VSELs (**b**) and HSCs (**c**)

**Fig. 2:**
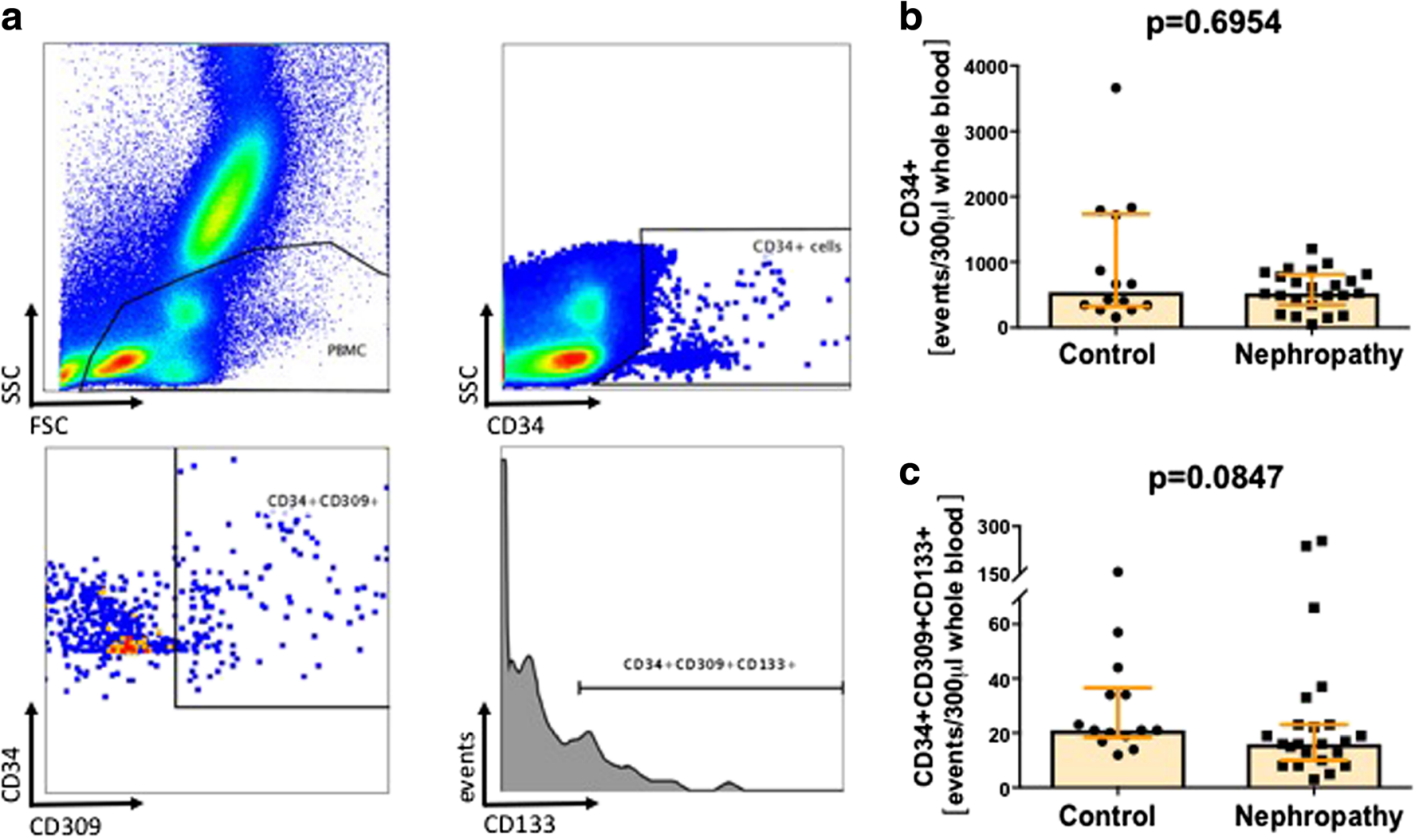
Flow cytometry analyses of total CD34+ cells and EPCs. Representative gating strategy for enumeration of total CD34+ cells and EPCs (CD34 + CD309 + CD133+) in peripheral blood (**a**). Summary of flow cytometry analyses of total CD34+ cells (**b**) and EPCs (**c**)

**Fig. 3:**
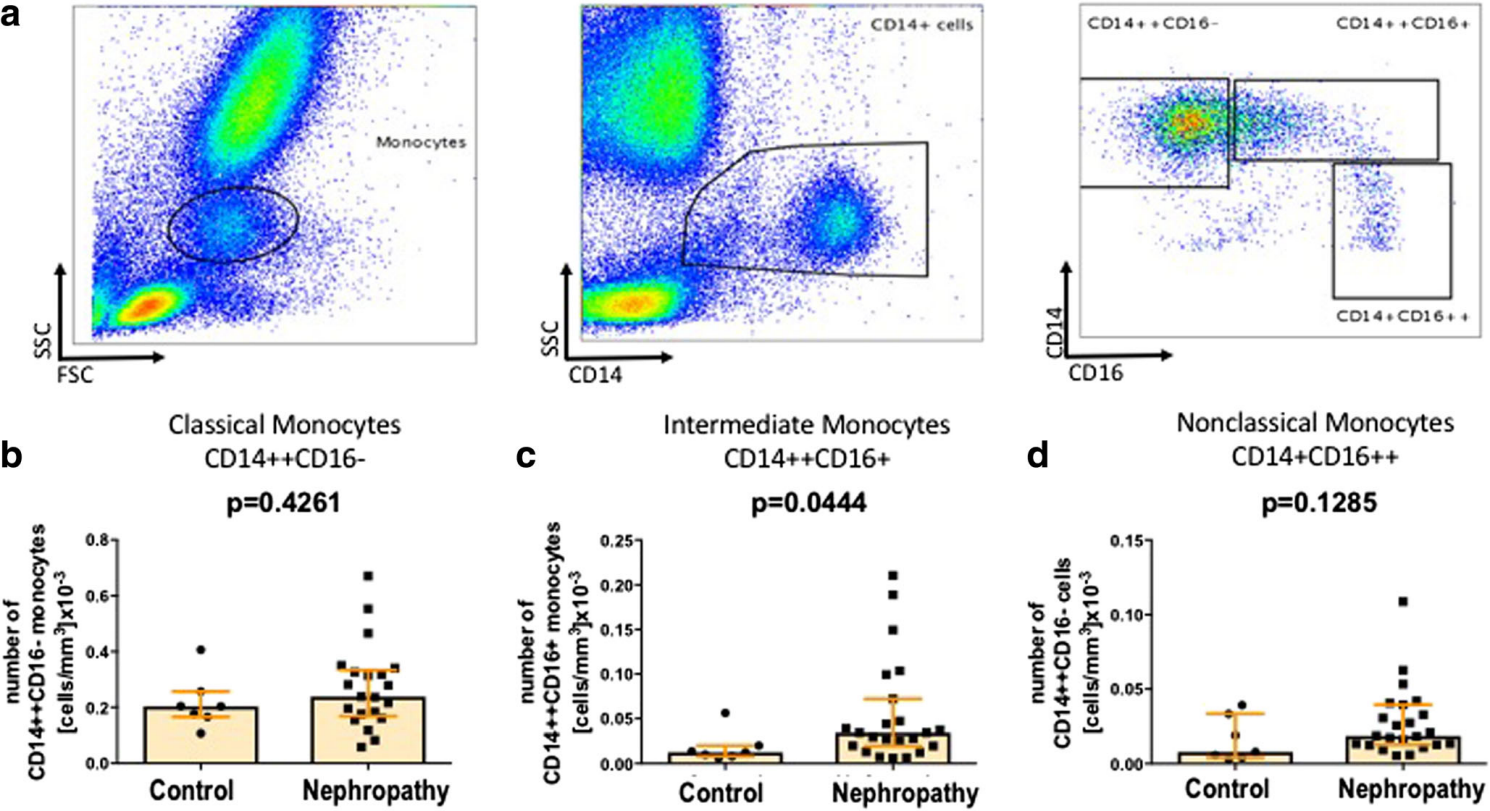
Elevated numbers of circulating intermediate (CD14++CD16+) monocytes in IgA nephropathy patients. Representative gating strategy for flow cytometric enumeration of different monocyte subsets (**a**). Summary of flow cytometry analyses of (**b**) classical (CD14++CD16-), (**c**) intermediate (CD14++CD16+) and non-classical (CD14 + CD16++) monocytes

600 μl (for VSELs and HSCs), 200 μl (for EPCs) and 100 μl (for monocytes) of fresh EDTA-anticoagulated whole blood was stained with a panel of monoclonal antibodies (mAbs; described in detail in Table 2) according to stain-then-lyse-then-wash protocol as described previously [20, 22, 23]. Briefly, samples were incubated in presence of mAbs for 30 min at room temperature in the dark. Next, 3 ml of FACS lysing solution (Becton Dickinson Bioscience) were added, followed by 15 min incubation at room temperature in the dark. Cells were washed twice in PBS and fixed with CellFix (BD) and analyzed by FACSCanto flow cytometer (BD). Appropriate fluorescence-minus-one (FMO) controls were applied for setting correct compensation and assuring proper gating. Obtained data were analyzed with FlowJo software (TreeStar inc.). Utilized gating strategies are presented on Fig. 1a for VSELs and HSCs; Fig. 2a for CD34+ cells and EPCs; and Fig. 3a for different monocyte subsets.

**Table 2:** Panel of monoclonal antibodies used for flow cytometry staining’s

Cellular Marker		Fluorochrome	Origin/isotype	Clone	Supplier
Anti-human lineage cocktail (lin2)	CD3	FITC	Mouse / IgG1	SK7	Becton Dickinson
	CD19	FITC	Mouse / IgG1	SJ25C1	Becton Dickinson
	CD20	FITC	Mouse / IgG1	L27	Becton Dickinson
	CD14	FITC	Mouse / IgG2b	MP9	Becton Dickinson
	CD56	FITC	Mouse / IgG1	NCAM16.2	Becton Dickinson
CD235a		FITC	Mouse / IgG2b	GA-R2	Becton Dickinson
CD45		PE	Mouse / IgG1	HI30	Becton Dickinson
CD133		APC	Mouse / IgG1	AC133	Miltenyi Biotec
CD14		PerCP	Mouse / IgG2b	MφP9	Becton Dickinson
CD16		FITC	Mouse / IgG1	NKP15	Becton Dickinson
CD34		FITC	Mouse / IgG1	8G12	Becton Dickinson
CD144		PE	Mouse / IgG1	55-7H1	Becton Dickinson
CD309		PE	Mouse / IgG	89106	Becton Disckinson

VSELs and HSC were gated as small (2-4um)/lin-/CD235a-/CD45-/CD133+ and small (2-4um)/lin-/CD235a-/CD45+/CD133+ cells, respectively. First, peripheral blood cells were visualized based on FSC and SSC signals. The 2-4um gate was set up according to size predefined beads. The threshold on FSC was set very low. Events from 2-4um gate were further analyzed for lineage markers and CD235a expression. Finally, lineage negative and CD235a negative events were gated and visualized on CD45/CD133 plot, and VSEL and HSC numbers were assessed.

EPCs were determined by CD34 + CD309 + CD133+ phenotype. First, all events were visualized based on FSC and SSC signals. Next, peripheral blood mononuclear cells (PBMC) were gated according to event size and granularity (lymphocyte and monocyte region was included, while debris and granulocyte region were excluded). Cells from PBMC gate were further analyzed for CD34 expression, and CD34+ cell numbers were determined. Next, CD34+ events were subsequently analyzed for CD309 expression. CD34 + CD309+ events were gated and visualized on CD133 histogram, and EPC numbers were determined.

Monocytes were gated according to both size and granularity on FSC/SSC plot and CD14 expression and granularity on CD14/SSC plot. Next, a boolean gate was generated as a sum of monocyte and CD14 + SSCint subset. Finally, frequencies of different monocyte subsets were assessed on the CD14/CD16 plot.

### Immunoassay

Soluble form of CD163 (sCD163), SDF-1, Angiopoietin-1 (Ang-1) and Angiopoietin-2 (Ang-2) levels in EDTA-plasma samples from patients with IgAN and normal donors were measured by means of commercially available enzyme-linked immunosorbent assays (DuoSet, R&D Systems) as previously described [24]. Protein levels were analyzed with automated light absorbance reader (LEDETEC96 system). Results were calculated according to standard curve by MicroWin 2000 Software.

### Statistics

Statistical analysis was carried out using GraphPad Prism 6 (GraphPad software). U Mann-Whitney test was used to assess differences between analyzed groups. Spearman correlation coefficient was used to determine correlations between variables. The differences were considered statistically significant at *p* < 0.05. The results are presented as median (interquartile range).

## Results

First, we aimed to quantitate the regenerative potential of IgAN patients by analyzing numbers of different circulating stem and progenitor cell subsets. We found that IgAN patients presented with significantly higher numbers of VSELs as compared to healthy controls (Fig. 1b). In contrast, the number of HSCs (Fig. 1c), total CD34+ cells (Fig. 2b) and EPCs (determined by CD34 + CD309 + CD133+ phenotype, Fig. 2c) in IgAN patients did not differ from controls. Moreover, we did not find significant correlations among frequencies of analyzed stem cell subsets and the biochemical parameters typical for kidney disease progression, namely: glomerular filtration rate (eGFR), protein extraction (uPCR), presence of hypertension and the doses of renin-angiotensin system inhibitors.

Next, we wished to analyze the differential distribution of different monocyte subsets with varying phenotypic and functional (including regenerative potential) properties, namely classical (CD14++CD16−), intermediate (CD14++CD16+) and non-classical (CD14 + CD16++) monocytes. Interestingly, in IgAN patients we found significantly higher numbers of intermediate but not classical and non-classical monocytes when compared to normal donors (Fig. 3). Again, we did not find significant correlations between different monocyte subsets and biochemical parameters of kidney disease progression.

**Fig. 4:**
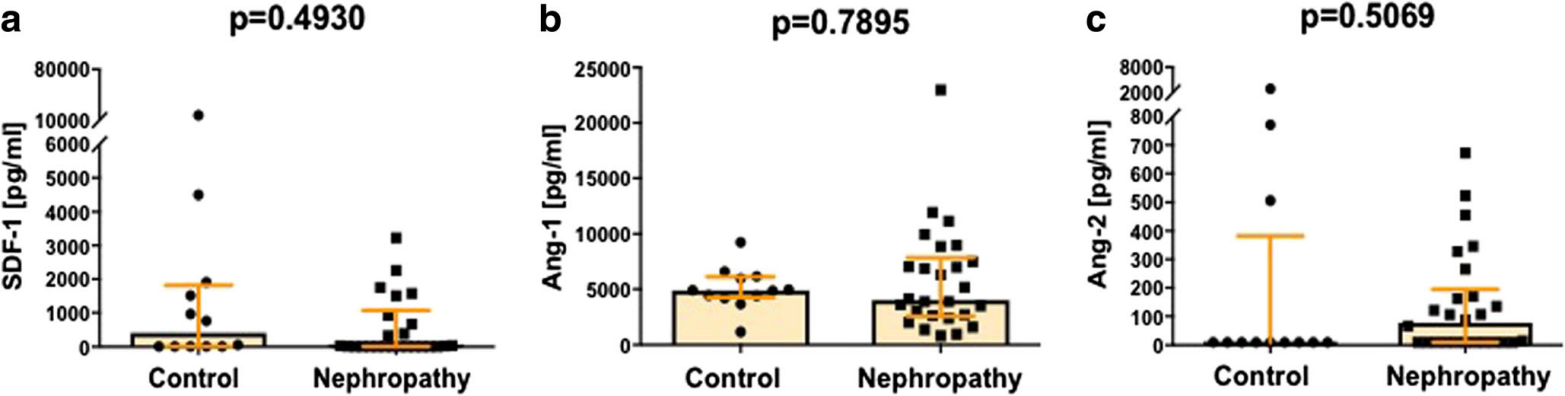
Serum levels of SDF-1 (**a**), Ang-1 (**b**) and Ang-2 (**c**) in IgA nephropathy patients and healthy controls

Having found increased numbers of VSELs and intermediate monocytes in peripheral blood of IgAN patients, we next set out to analyze whether their higher frequencies could have been associated with altered levels of already acknowledged chemotactic factors for VSELs and intermediate monocytes with proangiogenic potential, namely SDF-1 and Ang-1, Ang-2, respectively. However, levels of above mentioned chemotactic markers were similar in IgAN patients and healthy donors (Fig. 4). In addition, we did not find significant correlations between concentrations of these factors and numbers of analyzed stem cell subsets except for quite surprising significant (but still weak) correlation between SDF-1 levels and frequencies of intermediate monocytes (*r* = 0.0707; *p* = 0.0477).

**Fig. 5:**
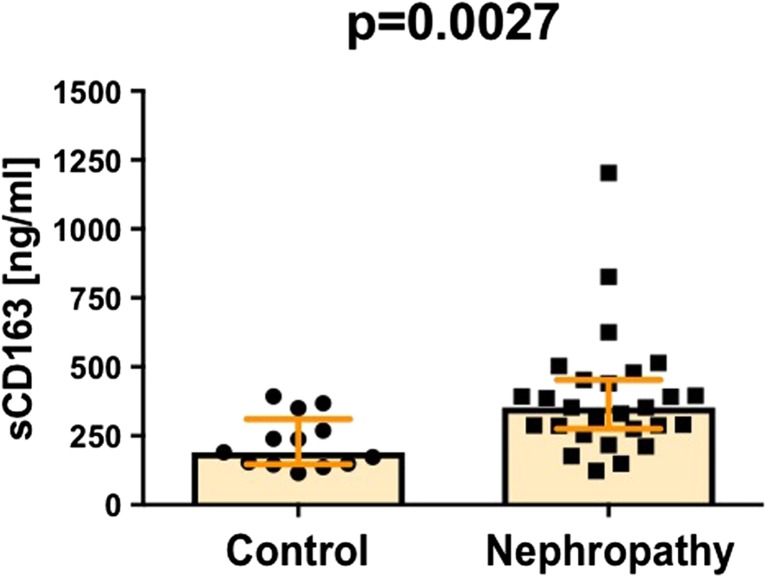
Elevated serum level of sCD163 in IgA nephropathy patients

Finally, we analyzed levels of soluble form of CD163, the protein that is most widely expressed by intermediate CD14++CD16+ monocytes [25]. Although, the function of soluble CD163 is not well recognized, however, detection of its elevated levels is widely considered the consequence of increased monocyte/macrophage activation [26]. Interestingly, we found significantly elevated levels of sCD163 in IgAN patients when compared to normal donors (Fig. 5). Somewhat surprisingly, sCD163 levels were negatively associated with SDF-1 levels (*r = −0*.3435; *p =* 0.0300) and positively correlated with Ang-2 levels (*r* = 0.4324; *p =* 0.0053).

## Discussion

In this study, we demonstrated that IgAN is associated with selective mobilization of VSELs but no other studied stem cell subsets. These findings point to two important explanations. First, chronic inflammation underlying IgAN can be related with activation of processes that are related with effective inhibition of stem cell subsets with very high regenerative potential such as HSCs or EPCs. Thus, further characterizing mechanisms accounting for inhibition of HSC- and EPC-related regeneration in IgAN could support the development of novel strategies aimed at decreasing the dynamics of kidney tissue damage. On the other hand, however, selective enhancement of VSELs in IgAN suggests unique role of this stem cell subset in the pathogenesis of this disorder. This notion could be also of clinical importance given very high regenerative potential of VSELs [2, 27, 28]. It remains to be established in further pre-clinical settings, whether naturally occurring mobilization of VSELs in IgAN can be further pharmacologically augmented in order to effectively counter-balance the effects of kidney injury.

Similarly, to our study, increased numbers of VSELs were observed previously in several other inflammatory disorders including myocardial infraction, pulmonary hypertension, COPD, stroke, active inflammatory bowel disease and cancer [2, 29–33]. It is believed, that spontaneous mobilization of VSELs into circulation depends on several factors released by injured or inflamed tissues, including SDF-1, hepatocyte growth factor (HGF), and leukemia inhibitory factor (LIF) signaling [31, 34–36]. Since, somewhat surprisingly, in this study we found no association between VSEL numbers and SDF-1 levels, we hypothesize that in IgAN settings, VSELs mobilization might be induced in SDF-1 independent manner.

Local excessive immune responses observed in many degenerative and autoimmune disorders, including IgAN cause considerable damage to tissues and may significantly affect proper organ function. These pro-inflammatory responses (related for example with IgA-secreting B cells) are expected to be counteracted by cells with anti-inflammatory properties, including M2 macrophages, regulatory T cells, tolerogenic dendritic cells and mast cells [18, 37–40]. Importantly, several reports indicated increased numbers of IgA-containing B cells, IgA-specific T-helper cells and decreased activity of regulatory T cells in IgAN patients [41–43]. To date, however, no studies in IgAN patients were assessing these monocyte subsets that are known to play regulatory and regenerative functions. Here, in our group of IgAN patients, we found significantly increased numbers of circulating intermediate CD14++CD16+ monocytes. Interestingly, intermediate CD14++CD16+ monocytes were shown, by our and other groups, to possess reparatory and proangiogenic potential [20, 24, 44, 45]. In fact, these cells may produce high amounts of anti-inflammatory cytokines and represent predominant population of circulating pro-angiogenic (Tie-2 expressing) leukocytes [44, 46, 47]. To date, increased numbers of pro-angiogenic/reparatory monocytes were reported in several inflammatory and degenerative disorders including cancer [24]. In these conditions, intermediate monocytes were considered precursors of anti-inflammatory M2-macrophage [24, 48]. Therefore, increased frequencies of intermediate CD14++CD16+, but not non-classical CD14++CD16- monocytes observed in this study in IgAN patients, may reflect their enhanced maturation towards M2-like anti-inflammatory cells. That hypothesis can be partially supported by our detection of increased plasma levels of sCD163 in IgAN patients. Since the expression of CD163 may be upregulated by anti-inflammatory signaling, CD163 is recognized as a marker of activation of anti-inflammatory M2 macrophages [49–51]. Importantly, soluble form of CD163 is released into circulation by proteolytic cleavage of extracellular part of the protein [26]. Thus, increased levels of sCD163 observed here, might be a consequence of local kidney-related activation of alternatively activated M2 macrophages. However, it is not certain whether M2 macrophages can necessarily play only anti-inflammatory and reparatory roles in IgAN. Recent reports indicated that CD163+ macrophages were selectively localized at sites of glomerular fibrinoid necrosis and were involved in crescent disease, acute tubular injury and glomerular lesions of IgAN [49]. Moreover, Gutiérrez and collaborators indicated that CD163 positive macrophages infiltration determine long-term renal outcome in macro hematuria-induced acute kidney injury of IgAN [52]. Furthermore, Endo and collaborators found that urinary sCD163 may serve as a biomarker of glomerular inflammation in lupus nephritis [53]. Notably, however, in this report we found no correlation between frequencies of precursors of M2 macrophages, CD14++CD16+ intermediate monocytes as well as sCD163 plasma levels and widely acknowledged disease progression parameters. Thus, our report adds to the current knowledge on the role of M2-like mononuclear phagocytes in IgAN by showing their increased activation but not relationship with the extent of tissue damage.

In summary, we demonstrated here that IgAN is characterized by selective augmentation of VSELs but not HSCs and EPCs as well as enhanced maturation of monocytes expressing M2-like and angiopoietic phenotype. These findings not only contribute to better understanding of the role of regulatory and regenerative mechanisms in IgAN but could also suggest several targets for development of novel therapies aimed at modulation of dysregulated stem cell or leukocyte functions.

## References

[1] Penfold RS, Prendecki M, McAdoo S, Tam FW (2018). Primary IgA nephropathy: Current challenges and future prospects. International Journal of Nephrology Renovascular Disease.

[2] Ratajczak MZ, Marycz K, Poniewierska-Baran A, Fiedorowicz K, Zbucka-Kretowska M, Moniuszko M (2014). Very small embryonic-like stem cells as a novel developmental concept and the hierarchy of the stem cell compartment. Advances in Medical Sciences.

[3] Ratajczak MZ (2017). Why are hematopoietic stem cells so ‘sexy’? On a search for developmental explanation. Leukemia.

[4] Kale S, Karihaloo A, Clark PR, Kashgarian M, Krause DS, Cantley LG (2003). Bone marrow stem cells contribute to repair of the ischemically injured renal tubule. The Journal of Clinical Investigation.

[5] Poulsom R, Forbes SJ, Hodivala-Dilke K (2001). Bone marrow contributes to renal parenchymal turnover and regeneration. The Journal of Pathology.

[6] Imasawa T, Utsunomiya Y, Kawamura T (2001). The potential of bone marrow-derived cells to differentiate to glomerular mesangial cells. Journal of the American Society of Nephrology.

[7] Ito T, Suzuki A, Okabe M, Imai E, Hori M (2001). Application of bone marrow-derived stem cells in experimental nephrology. Experimental Nephrology.

[8] Hayakawa M, Ishizaki M, Hayakawa J (2005). Role of bone marrow cells in the healing process of mouse experimental glomerulonephritis. Pediatric Research.

[9] Ikarashi K, Li B, Suwa M (2005). Bone marrow cells contribute to regeneration of damaged glomerular endothelial cells. Kidney International.

[10] Duffield JS, Park KM, Hsiao LL (2005). Restoration of tubular epithelial cells during repair of the postischemic kidney occurs independently of bone marrow-derived stem cells. The Journal of Clinical Investigation.

[11] Lin F, Moran A, Igarashi P (2005). Intrarenal cells, not bone marrow-derived cells, are the major source for regeneration in postischemic kidney. The Journal of Clinical Investigation.

[12] Zhang DW, Qiu H, Mei YM, Fu H, Zheng HG (2015). Repair effects of umbilical cord mesenchymal stem cells on podocyte damage of IgA nephropathy. Journal of Biological Regulators and Homeostatic Agents.

[13] Aggarwal S, Pittenger MF (2005). Human mesenchymal stem cells modulate allogeneic immune cell responses. Blood.

[14] Ohtaki H, Ylostalo JH, Foraker JE (2008). Stem/progenitor cells from bone marrow decrease neuronal death in global ischemia by modulation of inflammatory/immune responses. Proceedings of the National Academy of Sciences of the United States of America.

[15] Goerke SM, Obermeyer J, Plaha J, Stark GB, Finkenzeller G (2015). Endothelial progenitor cells from peripheral blood support bone regeneration by provoking an angiogenic response. Microvascular Research.

[16] Braza F, Dirou S, Forest V (2016). Mesenchymal stem cells induce suppressive macrophages through phagocytosis in a mouse model of asthma. Stem Cells.

[17] Jiang XX, Zhang Y, Liu B (2005). Human mesenchymal stem cells inhibit differentiation and function of monocyte-derived dendritic cells. Blood.

[18] Eljaszewicz A, Wiese M, Helmin-Basa A (2013). Collaborating with the enemy: Function of macrophages in the development of neoplastic disease. Mediators of Inflammation.

[19] Coffelt SB, Tal AO, Scholz A (2010). Angiopoietin-2 regulates gene expression in TIE2-expressing monocytes and augments their inherent proangiogenic functions. Cancer Research.

[20] Eljaszewicz A, Sienkiewicz D, Grubczak K (2016). Effect of periodic granulocyte colony-stimulating factor administration on endothelial progenitor cells and different monocyte subsets in pediatric patients with muscular dystrophies. Stem Cells International.

[21] Florez-Sampedro L, Song S, Melgert BN (2018). The diversity of myeloid immune cells shaping wound repair and fibrosis in the lung. Regeneration (Oxf).

[22] Zbucka-Kretowska M, Eljaszewicz A, Lipinska D (2016). Effective mobilization of very small embryonic-like stem cells and hematopoietic stem/progenitor cells but not endothelial progenitor cells by follicle-stimulating hormone therapy. Stem Cells International.

[23] Moniuszko M, Kowal K, Zukowski S, Dabrowska M, Bodzenta-Lukaszyk A (2008). Frequencies of circulating CD4+CD25+CD127low cells in atopics are altered by bronchial allergen challenge. European Journal of Clinical Investigation.

[24] Moniuszko M, Bodzenta-Lukaszyk A, Kowal K, Lenczewska D, Dabrowska M (2009). Enhanced frequencies of CD14++CD16+, but not CD14+CD16+, peripheral blood monocytes in severe asthmatic patients. Clinical Immunology.

[25] Møller HJ (2012). Soluble CD163. Scandinavian Journal of Clinical and Laboratory Investigation.

[26] Bhartiya D, Shaikh A, Anand S (2016). Endogenous, very small embryonic-like stem cells: critical review, therapeutic potential and a look ahead. Human Reproduction Update.

[27] Guerin CL, Rossi E, Saubamea B (2017). Human very small embryonic-like cells support vascular maturation and therapeutic revascularization induced by endothelial progenitor cells. Stem Cell Reviews.

[28] Guerin CL, Loyer X, Vilar J (2015). Bone-marrow-derived very small embryonic-like stem cells in patients with critical leg ischaemia: Evidence of vasculogenic potential. Thrombosis and Haemostasis.

[29] Wojakowski W, Tendera M, Kucia M (2009). Mobilization of bone marrow-derived Oct-4+ SSEA-4+ very small embryonic-like stem cells in patients with acute myocardial infarction. Journal of the American College of Cardiology.

[30] Marlicz W, Zuba-Surma E, Kucia M, Blogowski W, Starzynska T, Ratajczak MZ (2012). Various types of stem cells, including a population of very small embryonic-like stem cells, are mobilized into peripheral blood in patients with Crohn’s disease. Inflammatory Bowel Diseases.

[31] Marlicz W, Sielatycka K, Serwin K (2016). Effect of colorectal cancer on the number of normal stem cells circulating in peripheral blood. Oncology Reports.

[32] Guerin CL, Blandinières A, Planquette B (2017). Very small embryonic-like stem cells are mobilized in human peripheral blood during hypoxemic COPD exacerbations and pulmonary hypertension. Stem Cell Reviews.

[33] Ratajczak MZ, Zuba-Surma E, Kucia M, Reca R, Wojakowski W, Ratajczak J (2006). The pleiotropic effects of the SDF-1-CXCR4 axis in organogenesis, regeneration and tumorigenesis. Leukemia.

[34] Chen ZH, Lv X, Dai H (2015). Hepatic regenerative potential of mouse bone marrow very small embryonic-like stem cells. Journal of Cellular Physiology.

[35] Gharib SA, Dayyat EA, Khalyfa A (2010). Intermittent hypoxia mobilizes bone marrow-derived very small embryonic-like stem cells and activates developmental transcriptional programs in mice. Sleep.

[36] Idzkowska, E., Eljaszewicz, A., Miklasz, P., Musial, W. J., Tycinska, A. M., & Moniuszko, M. (2015). The role of different monocyte subsets in the pathogenesis of atherosclerosis and acute coronary syndromes. *Scandinavian Journal of Immunology*. 10.1111/sji.12314 25997925

[37] Josefowicz SZ, Lu LF, Rudensky AY (2012). Regulatory T cells: mechanisms of differentiation and function. Annual Review of Immunology.

[38] Steinman RM, Hawiger D, Nussenzweig MC (2003). Tolerogenic dendritic cells. Annual Review of Immunology.

[39] Nienartowicz A, Sobaniec-Łotowska ME, Jarocka-Cyrta E, Lemancewicz D (2006). Mast cells in neoangiogenesis. Medical Science Monitor.

[40] Harper SJ, Allen AC, Pringle JH, Feehally J (1996). Increased dimeric IgA producing B cells in the bone marrow in IgA nephropathy determined by in situ hybridisation for J chain mRNA. Journal of Clinical Pathology.

[41] Sakai H, Endoh M, Tomino Y, Nomoto Y (1982). Increase of IgA specific helper T alpha cells in patients with IgA nephropathy. Clinical and Experimental Immunology.

[42] Sakai H, Nomoto Y, Arimori S (1979). Decrease of IgA-specific suppressor T cell activity in patients with IgA nephropathy. Clinical and Experimental Immunology.

[43] Eljaszewicz A, Jankowski M, Gackowska L (2012). Gastric cancer increase the percentage of intermediate (CD14++CD16+) and nonclassical (CD14+CD16+) monocytes. Central European Journal of Immunology.

[44] De Palma M, Murdoch C, Venneri MA, Naldini L, Lewis CE (2007). Tie2-expressing monocytes: regulation of tumor angiogenesis and therapeutic implications. Trends in Immunology.

[45] De Palma M, Venneri MA, Galli R (2005). Tie2 identifies a hematopoietic lineage of proangiogenic monocytes required for tumor vessel formation and a mesenchymal population of pericyte progenitors. Cancer Cell.

[46] Skrzeczyńska-Moncznik J, Bzowska M, Loseke S, Grage-Griebenow E, Zembala M, Pryjma J (2008). Peripheral blood CD14high CD16+ monocytes are main producers of IL-10. Scandinavian Journal of Immunology.

[47] Murdoch C, Tazzyman S, Webster S, Lewis CE (2007). Expression of Tie-2 by human monocytes and their responses to angiopoietin-2. Journal of Immunology.

[48] Eljaszewicz A, Gackowska L, Kubiszewska I (2010). Macrophage activity in tumour development. Wspolczesna Onkologia-Contemporary Oncology.

[49] Li J, Liu CH, Gao B, Xu DL (2015). Clinical-pathologic significance of CD163 positive macrophage in IgA nephropathy patients with crescents. International Journal of Clinical and Experimental Medicine.

[50] Van Gorp H, Delputte PL, Nauwynck HJ (2010). Scavenger receptor CD163, a Jack-of-all-trades and potential target for cell-directed therapy. Molecular Immunology.

[51] Etzerodt A, Moestrup SK (2013). CD163 and inflammation: biological, diagnostic, and therapeutic aspects. Antioxidants & Redox Signal.

[52] Gutiérrez E, Egido J, Rubio-Navarro A (2012). Oxidative stress, macrophage infiltration and CD163 expression are determinants of long-term renal outcome in macrohematuria-induced acute kidney injury of IgA nephropathy. Nephron. Clinical Practice.

[53] Endo N, Tsuboi N, Furuhashi K (2016). Urinary soluble CD163 level reflects glomerular inflammation in human lupus nephritis. Nephrology, Dialysis, Transplantation.

